# Transcriptomic Analysis Reveals the Role of *AhERN1* in Peanut Nodulation

**DOI:** 10.3390/plants15121798

**Published:** 2026-06-11

**Authors:** Yue Wu, Jing Chen, Yan Ren, Guanchu Zhang, Qiangbo Liu, Yiteng Xu, Xue Zhang, Lijun Wu, Zhichao Lu, Hongfeng Wang

**Affiliations:** 1 Shandong Key Laboratory of Peanut Breeding, Shandong Peanut Research Institute, Qingdao 266100, China; wuyueswsw@126.com (Y.W.); chenjinghss@163.com (J.C.); cangry@sohu.com (Y.R.); guanchuzhang@126.com (G.Z.); wljd126@126.com (L.W.); 2 National Key Laboratory of Wheat Improvement, College of Life Sciences, Shandong Agricultural University, Tai’an 271018, China; liuqiangbo@sdau.edu.cn; 3 The Key Laboratory of Plant Development and Environmental Adaptation Biology, Ministry of Education, School of Life Sciences, Shandong University, Qingdao 266237, China; xuyiteng@sdu.edu.cn (Y.X.); lzcsdhz@163.com (Z.L.); 4 Advanced Medical Research Institute, Cheeloo College of Medicine, Shandong University, Jinan 250012, China; xuezhang@sdu.edu.cn

**Keywords:** peanut, transcriptomic analysis, ERN1, nodule development

## Abstract

Legume–rhizobium symbiosis represents a crucial biological nitrogen fixation system. The AP2/ERF transcription factor ERN1 plays a vital role in nodulation of model legumes; however, its function in peanut (*Arachis hypogaea*), a typical crack-entry infection legume, remains unclear. To explore this, we performed transcriptome sequencing of peanut roots at 3 days post-inoculation (dpi) with rhizobium. Gene ontology (GO) and Kyoto Encyclopedia of Genes and Genomes (KEGG) enrichment analyses showed that differentially expressed genes (DEGs) were mainly enriched in DNA-binding transcription factor activity, plant–pathogen interaction, and plant hormone signal transduction pathways. The most strongly up-regulated gene was *AhERN1*, which was highly expressed in peanut roots and nodules. Subcellular localization indicated that AhERN1 was a nuclear-localized protein, and yeast transcriptional activation assays confirmed that AhERN1 functions as a transcriptional activator relying on its C-terminal domain. Furthermore, hairy root overexpression of *AhERN1* significantly increased the number of peanut nodules. Collectively, these results reveal that *AhERN1* acts as a positive regulator to promote rhizobium-induced nodule development in peanut, providing new insights into the regulatory mechanism of nodulation in dalbergoid legumes.

## 1. Introduction

Legume–rhizobium symbiosis is one of the most efficient biological nitrogen fixation systems in nature, enabling leguminous plants to convert atmospheric nitrogen into ammonia via specialized root organs called nodules. This symbiotic interaction not only shapes the global nitrogen cycle but also reduces the dependence of agricultural production on chemical fertilizers, making it a core research target for sustainable agriculture [1]. The establishment of legume–rhizobium symbiosis is a precisely regulated process involving signal recognition, calcium signaling oscillation, transcriptional regulation, and organ morphogenesis [2]. Over the past two decades, forward and reverse genetics, comparative genomics, and cell biology studies in model legumes *Medicago truncatula* and *Lotus japonicus* have uncovered the core signaling pathways and key regulatory modules underlying symbiotic nodulation.

The symbiotic dialog initiates with the secretion of flavonoids by legume roots, which induces rhizobia to synthesize and secrete Nod factors (NFs) [3]. NFs are perceived by LysM domain-containing receptor kinases on the root epidermal cell membrane, including LjNFR1/LjNFR5 and MtLYK3/MtNFP, which act as primary receptors for symbiotic signal recognition and determine the host specificity of rhizobia infection [4,5,6,7]. Downstream of NF perception, the common symbiotic signaling pathway (CSSP) is activated, with perinuclear calcium spiking as a hallmark cellular response. Core components of CSSP include the leucine-rich repeat receptor-like kinase LjSymRK/MtDMI2 [8,9], nuclear envelope-localized cation channels LjPOLLUX/LjCASTOR/MtDMI1 [10,11,12], nucleoporins LjNUP85/LjNUP133 [13,14], and calcium/calmodulin-dependent protein kinase LjCCaMK/MtDMI3 [15,16,17]. LjCCaMK/MtDMI3 interacts with and phosphorylates LjCYCLOPS/MtIPD3, a key transcriptional activator that triggers downstream nodulation genes [18,19,20]. Downstream of the CCaMK-CYCLOPS complex, nodulation-specific GRAS family transcription factors NSP1 and NSP2 form a heterodimer to regulate the expression of early nodulin genes, and mutations in *NSP1* or *NSP2* completely block NF-induced gene expression and nodule formation [21,22,23]. Recent studies have shown that NSP1 can be phosphorylated by GSK3 kinase to inhibit nodulation, revealing a new layer of post-translational regulation [24]. The NSP1-NSP2 complex directly activates the expression of *NIN*, a master transcription factor that coordinates rhizobial infection and nodule primordium initiation [22,23].

NIN acts as a regulatory hub integrating multiple signals to orchestrate the entire symbiotic process [25,26,27,28]. NIN promotes infection thread growth and rhizobia colonization, while negatively regulating excessive infection to maintain symbiotic balance [29,30]. NIN also regulates CLE and CEP peptide signals to mediate the autoregulation of nodulation [31]. In the pericycle, NIN initiates nodule primordium formation by activating *LBD16* [32,33]. Notably, some dalbergoid legumes such as peanut retain a vestigial, less-efficient form of NF-independent symbiosis and an NIN-independent crack entry, reflecting the evolutionary plasticity of symbiotic mechanisms [34,35]. However, NIN plays a conserved role in peanut nodule organogenesis [34,36]. In root epidermal cells, AP2/ERF transcription factors ERN1, ERN2, and ERN3 specifically bind to the NF-box cis-element of early nodulins such as *ENOD11* to fine-tune NF-induced gene expression [37,38,39]. ERN1 and ERN2 function as transcriptional activators, whereas ERN3 acts as a repressor, forming a precise regulatory switch [37]. The *ERN1* gene is a direct target of the CCaMK-CYCLOPS complex, linking calcium signaling to transcriptional activation [40].

Phytohormone signaling is indispensable for nodule organogenesis, with cytokinin serving as a core regulator [41]. The cytokinin receptor LjLHK1/MtCRE1 is required for cortical cell division and *NIN* expression; gain-of-function mutations in *LHK1* trigger spontaneous nodulation in the absence of rhizobia [42,43]. Cytokinin signaling acts downstream of NF signaling and upstream of NIN [44,45]. While auxin, gibberellin, brassinosteroid, and ethylene also modulate nodule development and number [46,47,48,49,50,51,52].

In recent years, studies have expanded the symbiotic regulatory network and revealed diverse infection modes such as root hair infection and crack-entry and lateral root base nodulation. In root hair infection species, such as *Medicago truncatula*, *MtERN1* plays important roles in nodule development. Peanut as a dalbergoid legume with a unique crack-entry infection mode, but the function of the *MtERN1* ortholog in peanut remains unclear. In this study, by transcriptomic analysis, several genes were identified that are involved in the early stage of peanut nodulation. Furthermore, functional characterization of *MtERN1* ortholog *AhERN1* revealed that *AhERN1* acts as a transcription activator and promotes rhizobium-induced nodule development in peanut.

## 2. Results

### 2.1. Transcriptome Analysis of Genes Involved in Peanut Nodulation

**Figure 1:**
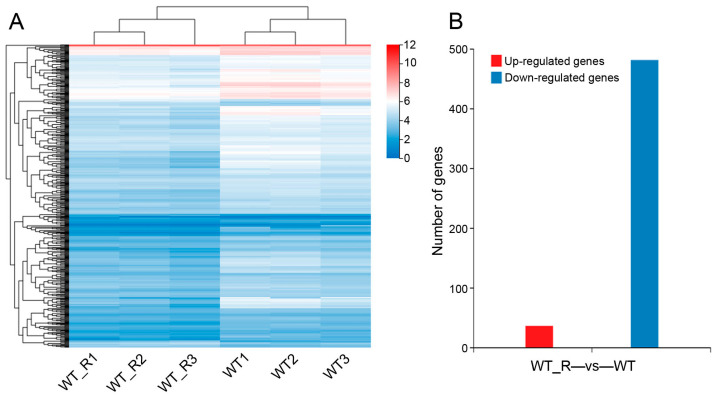
Differentially expressed genes in peanut inoculated and un-inoculated with rhizobia. (**A**) Heatmap representation of differentially expressed genes in root and root treated with rhizobia. (**B**) The number of differentially expressed genes identified in “treatment” compared to “control”.

To identify genes involved in the early stage of peanut nodulation, we conducted RNA sequencing for peanut whole roots after 3 dpi inoculation with rhizobium, named the “treatment”, and corresponding root parts without rhizobium infection, designated as the control. In total, six individual RNA samples were extracted with three replicates. Transcriptome analysis revealed that a total of 517 genes were differentially expressed between the “control” and the “treatment” (Figure 1A,B and Figure S1; Table S2). A total of 36 genes were up-regulated and 481 genes were down-regulated in response to the infection of rhizobia (Figure 1B).

### 2.2. Gene Ontology (GO) and Kyoto Encyclopedia of Genes and Genomes (KEGG) Analysis

**Figure 2:**
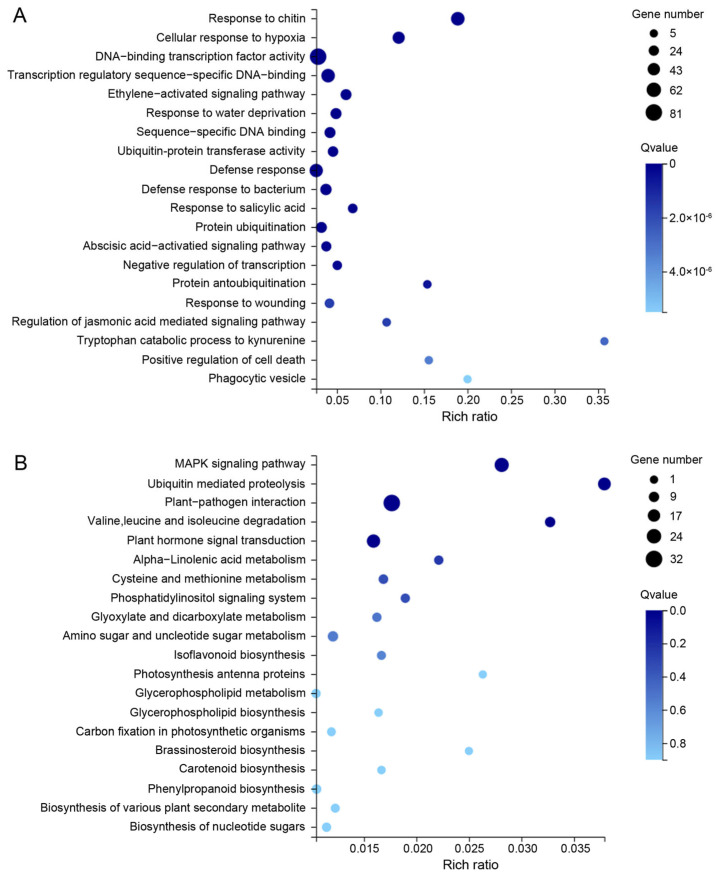
GO and KEGG enrichment analysis. (**A**) GO enrichment analysis of the 3 dpi treatment compared with the control. The horizontal axis indicates the gene-rich ratio, defined as the proportion of significantly enriched genes in a GO term relative to the total number of DEGs. The vertical axis represents significantly enriched GO terms. (**B**) KEGG enrichment analysis of the 3 dpi treatment compared with the control. The horizontal axis corresponds to the gene-rich ratio. The vertical axis displays the names of enriched KEGG pathways. The size of the bubble represents the number of DEGs annotated to a GO or KEGG term.

GO enrichment analysis showed that, after 3 days of rhizobia infection, the enriched GO terms were associated with DNA-binding transcription factor, sequence-specific DNA binding, defense response, response to chitin, defense response to bacterium, protein ubiquitination, and response to plant hormone, such as salicylic acid and abscisic acid (Figure 2A), indicating rhizobia infection triggered peanut gene transcription and defense response.

KEGG enrichment analysis revealed that the most significantly enriched gene ontology term was the plant–pathogen interaction process, followed by plant hormone signal transduction, MAPK signaling pathway, plant hormone signaling transduction, multiple amino acid metabolism, ubiquitin-mediated proteolysis, and flavonoid biosynthesis (Figure 2B), suggesting that these processes play critical roles in regulating early peanut nodulation. In depth analysis, we found auxin, gibberellin, brassinosteroid, jasmonic acid, and ethylene were differentially expressed, indicating plant hormones are involved in rhizobia-induced symbiosis.

### 2.3. Expression Pattern of the AhERN1 Gene

Transcriptomic profiling showed *AhERN1* was the most strongly up-regulated gene after rhizobia infection (Table 1; Figure S2), which is essential for nodule formation in legumes, such as *Medicago truncatula* and *Lotus japonicus*. Furthermore, the representative DEGs identified by transcriptome analysis were verified by RT-qPCR (Figure S3).

**Figure 3:**
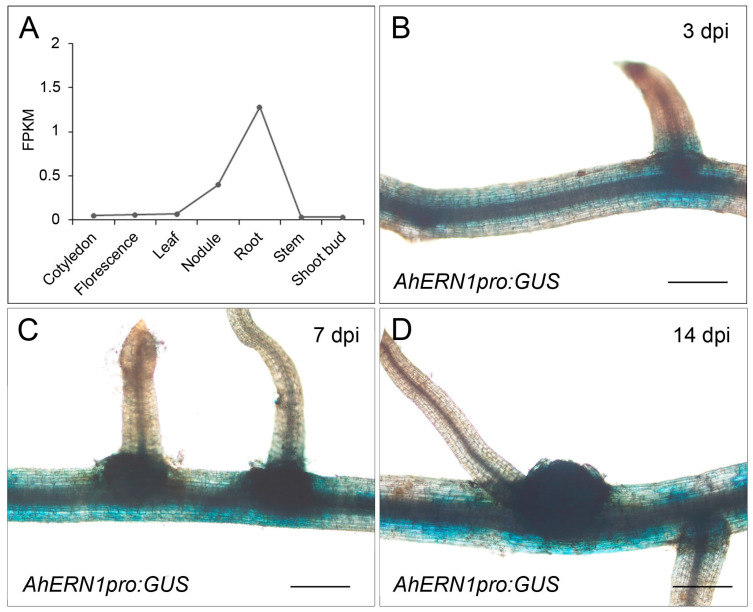
Expression patterns of the *AhERN1* gene in peanut. (**A**) Expression profile of the *AhERN1* gene in different organs of peanut. The expression data was constructed based on fragments per kilobase of transcript per million mapped reads (FPKM) values in the *A. hypogaea* transcriptome dataset (http://peanutgr.fafu.edu.cn accessed on 16 October 2025). (**B**–**D**) Promoter–GUS fusion analysis of *AhERN1* expression in hairy root transformed nodules at 3 dpi (**B**), 7 dpi (**C**), and 14 dpi (**D**). dpi, days post-inoculation. Scale bars, 1 mm in (**B**–**D**).

To explore the role of *AhERN1* in peanut nodule development, the expression patterns of *AhERN1* were analyzed. First, the expression profiles of the *AhERN1* gene in different organs and tissues of the cultivated peanut variety “Shitouqi” were analyzed using publicly available transcriptome sequencing data (http://peanutgr.fafu.edu.cn, accessed on 16 October 2025). The data showed that *AhERN1* was highly expressed in the root and nodule (Figure 3A), suggesting that *AhERN1* plays a crucial role in peanut nodule formation and development. Second, to analyze the expression patterns of *AhERN1* in nodules in more detail, the *proAhERN1:GUS* hairy root transgenic plants were obtained. Compared with the negative control empty vector (pBGWFS7) transformed roots (Figure S4), GUS staining analysis showed that *AhERN1* was expressed in nodules and roots (Figure 3B–D). These results demonstrate that *AhERN1* is involved in peanut nodule formation.

### 2.4. AhERN1 Encodes a Transcriptional Activator

**Figure 4:**
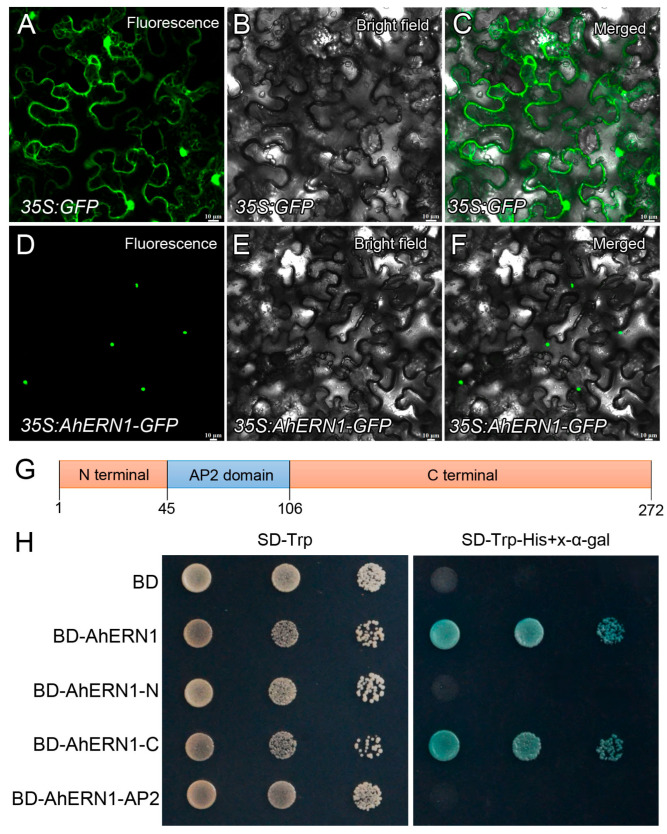
Subcellular localization and transcriptional activity of AhERN1. (**A**–**C**) The subcellular localization of the free GFP protein. (**D**–**F**) The subcellular localization of the AhERN1-GFP fusion protein. (**G**) Schematic illustration of the AhERN1 protein. (**H**) Analysis of the transcriptional activity of AhERN1 in yeast cells.

To further characterize the possible role of *AhERN1* in peanut nodule development, subcellular localization analysis of the *AhERN1* gene was performed. *AhERN1* was fused with GFP and transformed into tobacco epidermal cells. Green fluorescence signal observation revealed that, compared with free GFP, which is located in the cell cytoplasm and cell nucleus (Figure 4A–C), *AhERN1* was located in the cell nucleus (Figure 4D–F), indicating that *AhERN1* encodes a nuclear-localized transcription factor.

Furthermore, the transcriptional activity of AhERN1 was analyzed. First, protein structure analysis showed that AhERN1 contains a N-terminal sequence, a conserved AP2 domain, and a C-terminal sequence (Figure 4G and Figure S5). To analyze the transcriptional activity of AhERN1, the CDS of the *AhERN1* gene was in-frame fused with the GAL4 DNA-binding domain (BD) to generate the AhERN1:BD vector. Transcriptional activation assay showed that, compared to the control transformation (BD), the yeast cell transforming with AhERN1:BD grew well on the -Trp-His deficient SD medium (Figure 4H), suggesting AhERN1 had transcriptional activation activity. Domain truncation analysis showed that the transcriptional activation activity of AhERN1 depends on its C-terminal (Figure 4H). These results suggest that AhERN1 may function as a transcriptional activator to regulate peanut nodule development.

### 2.5. AhERN1 Promotes Rhizobium-Induced Nodule Development in Peanut

**Figure 5:**
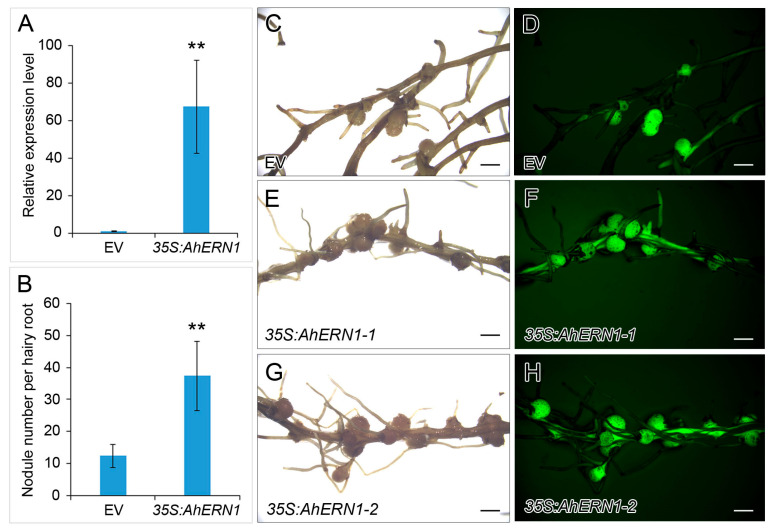
Phenotypic analysis of *AhERN1* overexpression nodules. (**A**) RT-qPCR analysis of the expression of the *AhERN1* gene in control (EV) and *AhERN1*-overexpressing nodules at 28 dpi. Values are the means and SDs of three sample replicates; ** *p* < 0.01. (**B**) Nodule number of control (EV) and hairy roots transformed with the *35S:AhERN1* vector. Values are the means and SDs of ten sample replicates; ** *p* < 0.01. (**C**–**H**) Representative stereoscopic bright field (**C**,**E**,**G**) and fluorescence images (**D**,**F**,**H**) of EV (**C**,**D**) and *35S:AhERN1* (**E**–**H**) nodulated hairy roots. Scale bars, 2 mm in (**C**–**H**).

To investigate the function of *AhERN1* in peanut nodulation, we overexpressed *AhERN1* under the control of the enhanced cauliflower mosaic virus (CaMV) 35S promoter in the shitouqi background. RT-qPCR analysis confirmed that the expression of *AhERN1* was up-regulated in *35S:AhERN1* hairy roots compared to the control (EV) (Figure 5A). At 28 dpi with *Bradyrhizobium* sp. HHPB1, the *AhERN1*-overexpressing hairy roots produced more nodules on their roots than EV (Figure 5B–H). The results demonstrated that *AhERN1* has a promotive effect on peanut nodulation. In order to evaluate the effect of activating *AhERN1* on host gene expression, we analyzed the expression of some symbiotic genes by RT-qPCR. In *35S:AhERN1* nodules, the expression levels of *AhNFR5* and *AhHK1* were up-regulated (Figure S6A,B), indicating that AhERN1 promotes symbiotic signaling and cytokinin signaling transduction. However, the transcript level of *AhNIN* did not change significantly in the control and *35S:AhERN1* nodules (Figure S6C), indicating that *AhNIN* may play a role in parallel with *AhERN1*. In addition, we found that *AhENOD40* was strongly induced in *35S:AhERN1* nodules (Figure S6D), indicating that *AhENOD40* is involved in peanut nodule development and is dependent on *AhERN1*. In future studies, obtaining *AhERN1* knockout/knockdown plants will provide direct genetic evidence that AhERN1 is a core regulator involved in peanut nodulation.

## 3. Discussion

Legume–rhizobium symbiosis supports efficient biological nitrogen fixation and greatly reduces reliance on synthetic nitrogen fertilizers, making it indispensable for sustainable agricultural systems. In model legumes including *Medicago truncatula* and *Lotus japonicus*, the AP2/ERF transcription factor ERN1 has been established as a key regulator of rhizobial infection and nodule organogenesis [37,38,40]. However, peanut (*Arachis hypogaea*) represents a distinct dalbergoid legume that utilizes a crack-entry infection strategy, which develops an NF-dependent or independent symbiosis [33]. The NIN signaling pathway is involved in peanut nodule organogenesis, but not in crack entry [34]. To date, the molecular function of ERN1 orthologs in peanut nodulation has remained largely uncharacterized. In this study, we performed transcriptomic analysis of peanut roots at 3 days post-inoculation (dpi) with rhizobium and systematically characterized *AhERN1*, revealing its essential role in nodule development.

Transcriptome sequencing identified 517 differentially expressed genes (DEGs) in response to early rhizobial inoculation, with 36 up-regulated and 481 down-regulated genes. GO enrichment analysis showed that DEGs were predominantly enriched in DNA-binding transcription factor activity, sequence-specific DNA binding, defense response, and response to chitin. These annotations indicate that transcriptional reprogramming and innate immune responses are rapidly activated during the early stages of peanut–rhizobium interaction. KEGG enrichment further highlighted significant enrichment in plant–pathogen interaction, plant hormone signal transduction, and MAPK signaling pathways, consistent with the dual activation of symbiotic signaling and basal defense during rhizobial colonization. It has been reported that plant hormones are involved in rhizobial symbiosis and nodule organ formation [53,54,55,56]. In our KEGG enrichment results, auxin signal components AUX/IAA and SAUR, gibberellin signal component DELLA, brassinosteroid signal component BRI1, jasmonic acid signal component MYC2, and ethylene signal components SIMKK and EBF1/2 were differentially expressed during peanut symbiosis. Notably, *AhERN1* was the most strongly up-regulated gene upon rhizobial infection, suggesting a central role in early nodulation signaling. Future application of single-cell and spatial transcriptomics will help resolve cellular heterogeneity and spatial transcriptional regulation during peanut crack-entry nodulation and further dissect the AhERN1-associated regulatory network [57].

Expression profiling confirmed that *AhERN1* was highly and specifically expressed in roots and nodules, with low transcript abundance in other organs. Promoter–GUS staining showed continuous expression of *AhERN1* in developing nodules at 3, 7, and 14 dpi, supporting a sustained function throughout nodule formation. This tissue-specific pattern mirrors that of *ERN1* genes in model legumes [38,39], suggesting evolutionary conservation in symbiotic regulation.

Subcellular localization revealed that the AhERN1-GFP fusion protein was exclusively localized in the nucleus, consistent with its predicted role as a transcription factor. Yeast transcriptional activation assays demonstrated that AhERN1 functions as a transcriptional activator, with the activation domain located in the C-terminal region. Truncated versions lacking the C-terminus failed to activate reporter gene expression, indicating that the C-terminal domain is essential for its transcriptional activity. These structural and functional features are conserved among ERN1 proteins across legume species.

In *Lotus japonicus* and *Medicago truncatula* root nodule symbiosis, rhizobia are striped by the root hair and enter the root cortex via infection threads (ITs). Loss-of-function mutants of MtERN1/LjERN1 orthologs are completely unable to initiate infection or nodule development [37,39,40,58]. In this study, functional analysis using hairy root overexpression showed that constitutive expression of *AhERN1* significantly increased nodule number at 28 dpi compared with empty vector controls. These results provide evidence that AhERN1 acts as a positive regulator of nodulation in peanut. However, constitutive 35S promoter-driven *AhERN1* expression may artificially alter developmental or hormonal processes unrelated to nodulation. In the future, the acquisition of *AhERN1* homologous gene-edited plants or tissue-specific expression of *AhERN1* will provide direct genetic evidence that *AhERN1* is a core regulator involved in peanut nodulation. In model legumes, ERN1 acts downstream of the CCaMK-CYCLOPS complex to regulate early nodulin genes [40]. Our findings reveal that AhERN1 retains conserved molecular characteristics (nuclear localization, transcriptional activation) and functions in nodulation even in legumes with NIN-independent crack-entry infection [34,35]. This discovery extends the functional scope of ERN1 to dalbergoid legumes and underscores the evolutionary flexibility of symbiotic signaling networks. The expression of *AhENOD40* was strongly induced in *35S:AhERN1* nodules, indicating that *AhENOD40* may act downstream of *AhERN1* to regulate peanut nodule development. Future research will focus on identifying direct interaction proteins and downstream targets of AhERN1 and dissecting its crosstalk with phytohormone signaling pathways, which will further refine the regulatory network governing peanut–rhizobium symbiosis.

## 4. Materials and Methods

### 4.1. Plant Materials and Growth Conditions

The cultivated peanut variety “Shitouqi” was used as the experimental material in this study. The seeds were planted into seedling pots (5 × 5 × 8 cm length, width, height) with sterilized vermiculite and grown in a growth chamber (day: 16 h, 25 °C; night: 8 h, 25 °C; relative humidity: 75%).

### 4.2. Subcellular Localization Analysis

To analyze the subcellular localization of AhERN1, the CDS of the *AhERN1* gene was amplified using specific primer pairs AhERN1-CDS-F and AhERN1-CDS-R (Supplementary Table S1). Then, the amplified PCR products were purified and subcloned into the *pENTR/D TOPO* vector to generate the *AhERN1:pENTR* vector. Finally, the subcellular localization analysis vector *35S:AhERN1-GFP* was generated by a gateway recombination reaction between the *pEarleyGate 103* and *AhERN1:pENTR* vectors. For tobacco subcellular localization analysis, 21-day-old *Nicotiana benthamiana* leaves were injected with *Agrobacterium* GV3101 (pSoup-p19) strain suspension with OD600 = 0.8 containing the *35S:AhERN1-GFP* plasmid. After incubation in the dark for 24 h and then in the light for 48 h, the tobacco leaves were observed. For fluorescent imaging, a Leica LSM 880 laser scanning confocal microscope was used. The 488 nm line of an argon laser was chosen for the green fluorescent protein (GFP) signal.

### 4.3. Promoter β-Glucuronidase (GUS) Staining Analysis

For *AhERN1* gene promoter expression pattern analysis, the 1867 bp promoter sequence of the *AhERN1* gene was amplified using gene-specific primer pairs AhERN1-Pro-F and AhERN1-Pro-R (Supplementary Table S1). Then, the promoter sequence of *AhERN1* was subcloned into the *pENTR/D TOPO* vector to generate the *AhERN1pro:pENTR* vector. Finally, the *AhERN1pro:GUS* destination vector was generated by a gateway recombination reaction between the gateway vectors *pBGWFS7* and *AhERN1pro:pENTR*. The empty vector *pBGWFS7* was used as a negative control.

### 4.4. Overexpression Analysis

For *AhERN1* gene overexpression analysis, first, the *AhERN1:pENTR* vector was recombined with the gateway vector *pB7WG2D* to generate the overexpressing vector *AhERN1:pB7WG2D.* Second, the *Agrobacterium rhizogenes* strain K599 containing the overexpressing vector *AhERN1:pB7WG2D* was hairy transformed into peanut. The empty vector (EV) *pB7WG2D* was used as a control. Due to the *pB7WG2D* vector containing an independent GFP marker, the nodules formed in the EV and *AhERN1*-overexpressing hairy roots displayed green fluorescence, and the number of nodules was counted at 28 dpi.

### 4.5. Hairy Root Transformation and Root Nodule Induction

For peanut hairy root transformation, the *Agrobacterium rhizogenes* strain K599 containing the plasmids was transformed into peanut hypocotyl. Briefly, healthy peanut seedlings at 7 days post-germination, with fully expanded cotyledons, were used for hairy root transformation. First, the primary root was removed using sterile scissors, leaving a 1 cm segment of the hypocotyl intact. Second, the wound site was thoroughly coated with *Agrobacterium rhizogenes* strain K599 harboring the corresponding vectors. Third, the seedlings whose roots were covered with *Agrobacterium rhizogenes* were directly inserted into moist sterilized vermiculite and grown in a growth chamber. After approximately four weeks of growth, the transgenic hairy roots could be visually identified. Throughout this period, vermiculite moisture was consistently maintained to ensure successful root induction.

For nodule induction, the peanut hairy roots were treated with 2 mL of commercial rhizobia strain suspension (*Bradyrhizobium* sp. HHPB1; Colony-Forming Units (CFUs) ≥ 3 million/mL; Anhui New Simon Biotech Co., Ltd., Hefei, China) and grown in a growth chamber.

### 4.6. Transcriptional Activity Analysis

The full length of the AhERN1 protein and the truncated fragments of AhERN1 (1–44 aa: N-terminal, 45–106 aa: AP2 domain, and 107–272 aa: C-terminal) were amplified using gene-specific primer pairs (Supplementary Table S1) and inserted into pGBKT7 and transformed into yeast (*Saccharomyces cerevisiae*) strain AH109. The empty vector pGBKT7 served as a negative control, and the successful transformants were selected on SD/-Trp-His medium supplemented with x-α-gal.

### 4.7. RNA Extraction and Reverse Transcription Quantitative PCR (RT-qPCR)

To analyze the relative expression levels of *AhERN1*, *AhNRF5*, *AhHK1*, *AhNIN*, and *AhENOD40* genes in the control (EV) and *AhERN1*-overexpressing hairy roots, each independent hairy root was collected after 28 days of inoculation with rhizobia. The nodules were taken off hairy roots and quickly stored in liquid nitrogen. Then, the total RNA of different samples was extracted using the TRIzol-RT Reagent (Molecular Research Center, Inc., Cincinnati, OH, USA) according to the manufacturer’s instructions. A total of 1.5 micrograms of total RNA from each sample was reverse-transcribed into cDNA using the HiScript IV All-in-One Ultra RT SuperMix Kit (Vazyme, Nanjing, China). The RT-qPCR analysis was performed on Bio-Rad CFX Connect^TM^ using 2X M5 HiPer Realtime PCR mix (Mei5bio, Beijing, China). The relative expression levels were calculated using the 2^−∆∆CT^ method. The *AhG6PD* gene was selected as the housekeeping gene for normalization [59].

### 4.8. RNA Sequencing

For RNA sequencing analysis, seedlings were grown on sterile vermiculite for one week. Then, the seedlings were treated with 2 mL of commercial rhizobia strain suspension (*Bradyrhizobium* sp. HHPB1; Colony-Forming Units (CFUs) ≥ 3 million/mL; Anhui New Simon Biotech Co., Ltd., Hefei, China). The control seedlings were treated with an equal volume of water. At 3 dpi, the whole roots were collected and quickly immersed in liquid nitrogen before total RNA extraction. The whole transcriptome sequencing was performed by the BGI center (Shenzhen, China). Differential expression analysis was performed using the DESeq2 R package. *p* < 0.05 was set as the threshold for significantly differentially expressed genes.

### 4.9. Statistical Analysis

All the data are expressed as the means ± standard deviations (SDs) using Excel 2021. GraphPad Prism 5.0 was used to map the final data, and differences among gene expression level and nodule number were examined by Student’s *t*-test (** *p* < 0.01).

## 5. Conclusions

This study identified AhERN1 as the most significantly up-regulated transcription factor during early peanut nodulation via transcriptome sequencing of rhizobium-inoculated roots. Expression analysis showed that *AhERN1* was specifically and highly expressed in peanut roots and nodules, implying a close association with symbiotic nodulation. Subcellular localization verified that AhERN1 was a nuclear-localized protein, and yeast transcriptional activation assays confirmed its function as a transcriptional activator dependent on the C-terminal domain. Furthermore, overexpression of *AhERN1* in peanut hairy roots significantly increased nodule number. Collectively, these results demonstrate that AhERN1 acts as a positive regulator to promote rhizobium-induced nodule development in peanut, a typical dalbergoid legume with a crack-entry infection pattern. Our findings expand the functional understanding of ERN1 family genes in legume nodulation and provide a valuable molecular target for genetic improvement of nitrogen fixation efficiency in peanut.

## Figures and Tables

**Table 1:** The top eight up-regulated genes in response to rhizobia infection.

Gene ID	log2 (WT_R/WT)	Qvalue (WT_R-vs-WT)	Description
AH06G22200.1	21.21800171	1.36577 × 10^−5^	ERF required for nodulation (ERN1)
AH12G03440.1	5.597113903	0.029563154	Putative disease resistance protein RGA4
AH16G44230.1	5.551477951	0.012860962	UDP-sugar pyrophosphorylase
AH12G36210.1	5.351363157	0.047003202	Protein fluG
AH19G05900.1	4.684950634	0.028193782	IAA-amino acid hydrolase ILR1-like 4
AH11G23790.1	4.402744108	0.049117480	Uncharacterized protein
AH07G21980.1	3.444614387	0.000479153	DEAD-box ATP-dependent RNA helicase
AH10G11060.1	3.274831022	0.015982975	Zinc finger domain-containing protein

## Data Availability

The original contributions presented in this study are included in the article and Supplementary Materials. Further inquiries can be directed to the corresponding author.

## Supplementary Materials

The following supporting information can be downloaded at https://www.mdpi.com/article/10.3390/plants15121798/s1: Figure S1. The principal component analysis (PCA) of transcriptome; Figure S2. Phylogenetic analysis of *ERN* genes in *A. hypogaea* (Ah), *A. thaliana* (At), *L. japonicus* (Lj) and *M. truncatula * (Mt); Figure S3. Representative DEGs identified by transcriptome analysis were verified by RT-qPCR; Figure S4. Empty vector (pBGWFS7) transformed roots as a negative control; Figure S5. Protein structure of AhERN1 predicted by Swiss-model; Figure S6. The expression levels of key symbiotic signaling pathway genes. RT-qPCR analyses of *AhNFR5* (A), *AhHK1* (B), *AhNIN* (C) and *AhENOD40* (D) transcripts was performed using total RNA samples extracted from control (EV) and *AhERN1*-overexpressing (*35S:AhERN1*) nodules at 28 dpi; Table S1. Primers used in this study; Table S2. Differentially expressed genes between control and treatment.

## Abbreviations

The following abbreviations are used in this manuscript:

ERN1	Ethylene Response Factor Required for Nodulation 1
NFR1/5	NOD FACTOR RECEPTOR 1/5
LYK3	LysM RECEPTOR KINASE 3
NFP	NOD FACTORPERCEPTION
CSSP	Common Symbiotic Signaling Pathway
SymRK	Symbiotic Receptor-like Kinase
NUP85/133	NUCLEAR PORE COMPLEX PROTEIN 85/133
CCaMK	Ca^2+^/CaM-dependent protein kinase
IPD3	INTERACTING PROTEIN OF DMI3
NSP1/2	NODULATION SIGNALING PATHWAY 1/2
NIN	NODULE INCEPTION
LBD16	LATERAL ORGAN BOUNDARIES (LOB)- DOMAIN PROTEIN 16
CLE	CLAVATA3/ESR-related (CLE) peptides
CEP	C-terminally Encoded Peptides
ENOD11/40	EARLY NODULIN11/40
HK1	Cytokinin Receptor Histidine Kinase 1
AUX/IAA	Auxin/Indole-3-acetic Acid
SAUR	Small Auxin Up-regulated RNA
BRI1	Brassinosteroid-insensitive 1
SIMKK	Stress-induced MAPK Kinase
EBF1/2	F-box Proteins EIN3-BINDING F-BOX 1/2
FPKM	Fragments Per Lilobase of Transcript Per Million Mapped Reads
