# Supplementary material for: Transcriptomic Analysis Reveals the Role of AhERN1 in Peanut Nodulation
Source: Plants (Basel). 2026 Jun 11;15(12):1798. doi: 10.3390/plants15121798 (PMC13306481; doi:10.3390/plants15121798)
Supplement: Supplementary file 1 [file plants-15-01798-s001.zip › Supplementary File—Revised.pdf]

Supplementary data

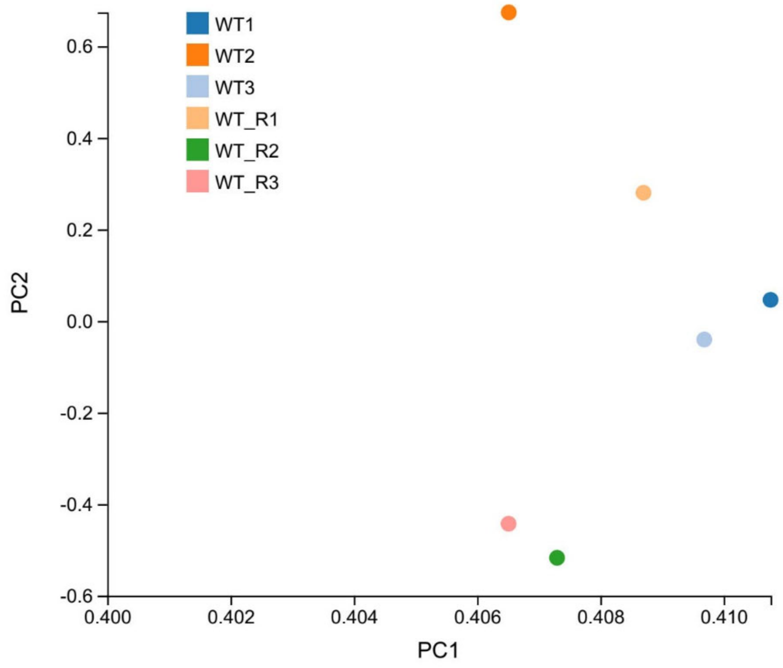

Figure S1. The principal component analysis (PCA) of transcriptome.

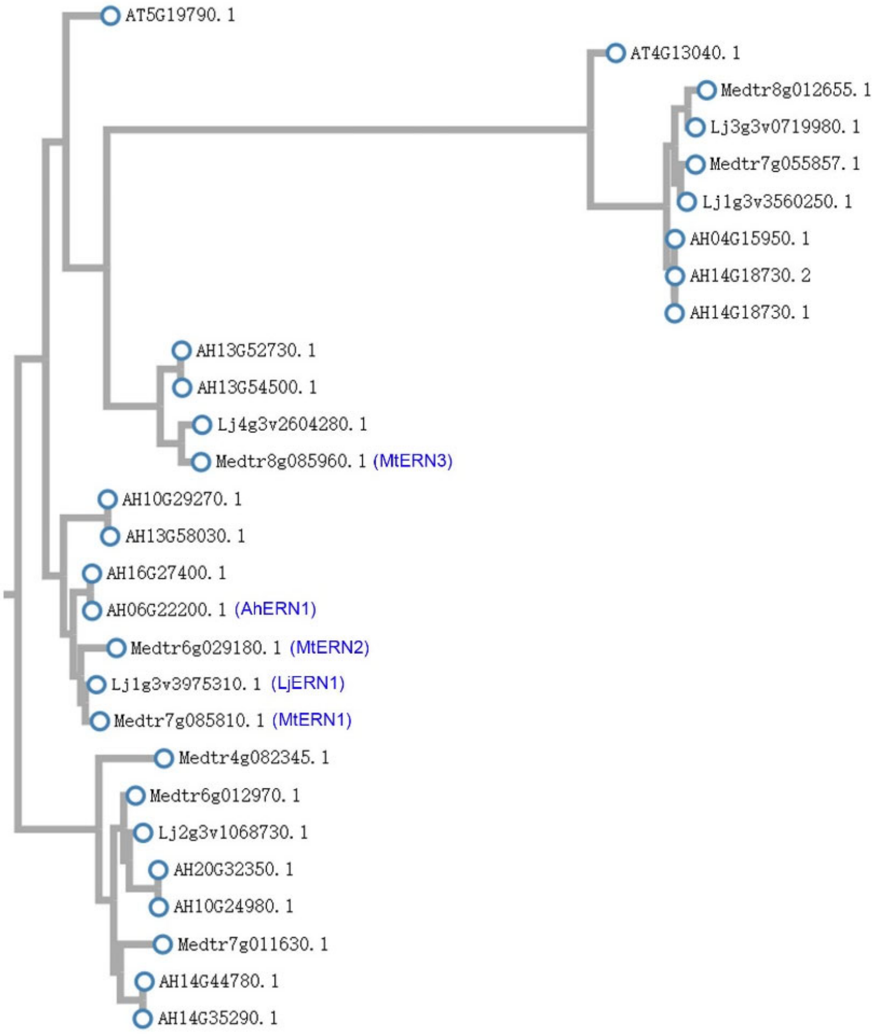

Figure S2. Phylogenetic analysis of *ERN* genes in *A. hypogaea* (Ah), *A. thaliana* (At), *L. japonicus* (Lj) and *M. truncatula* (Mt). The phylogenetic tree was constructed by online CLUSTALW website (https://www.genome.jp/tools-bin/clustalw).

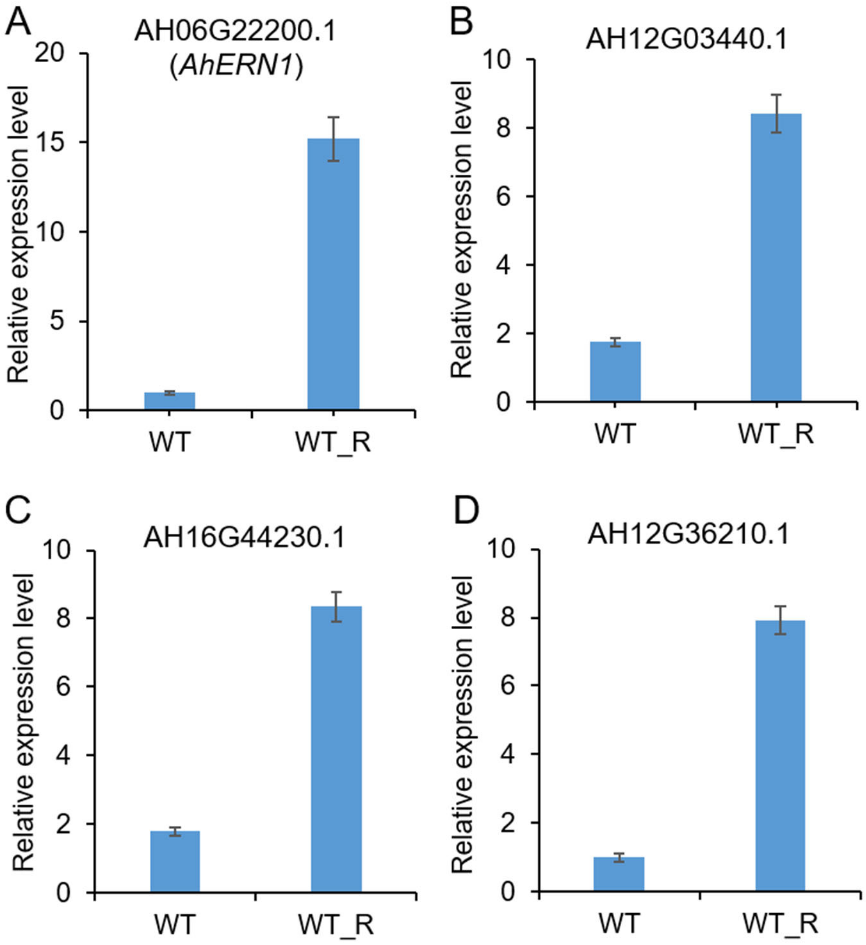

Figure S3. Representative DEGs identified by transcriptome analysis were verified by RT-qPCR.

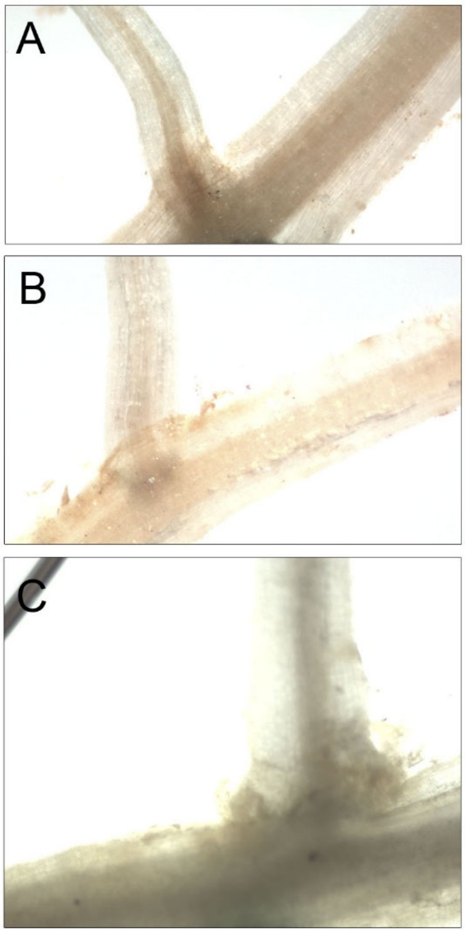

Figure S4. Empty vector (pBGWFS7) transformed roots as a negative control.

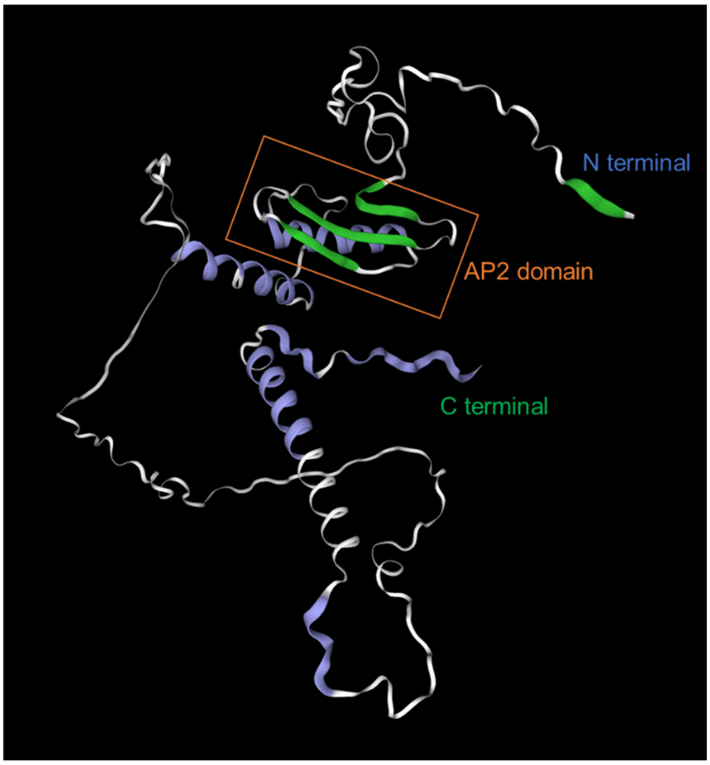

Figure S5. Protein structure of AhERN1 predicted by Swiss-model**.** The amino acid sequence framed by orange box is AP2 domain.

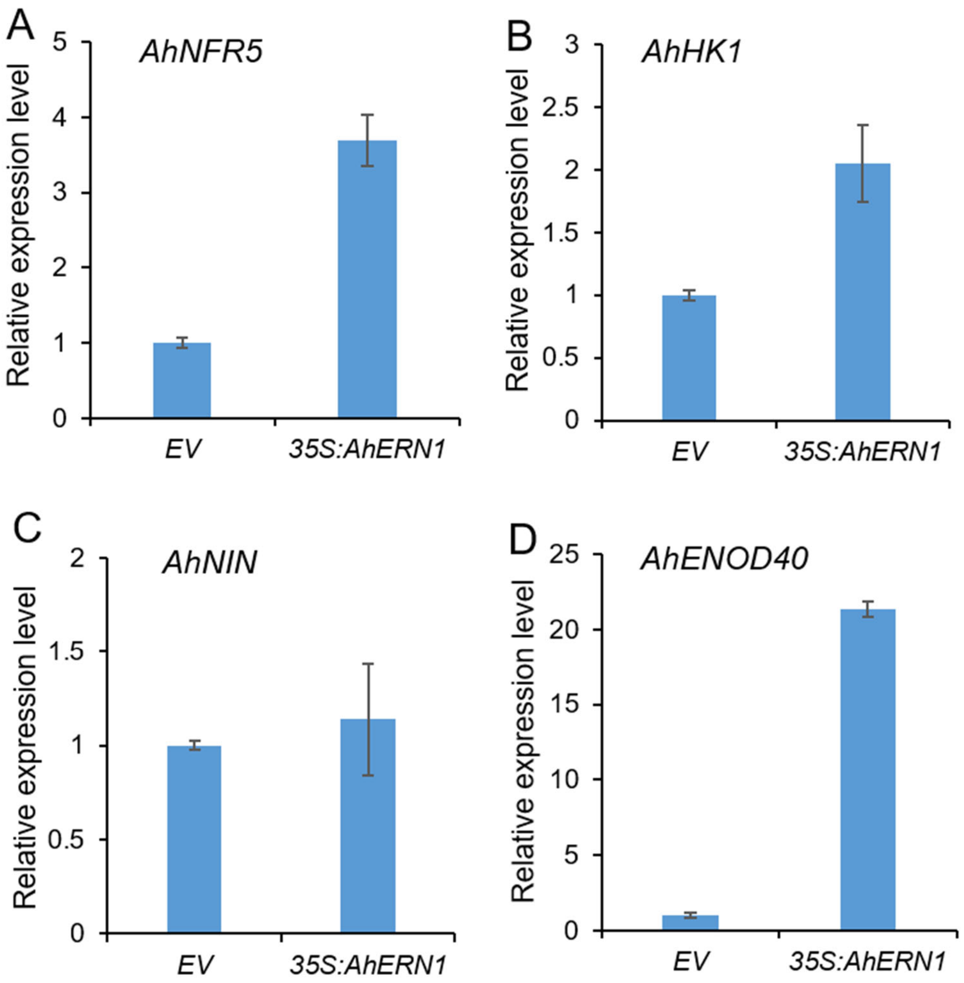

Figure S6. The expression levels of key symbiotic signaling pathway genes. RT-qPCR analyses of *AhNFR5* (A), *AhHK1* (B), *AhNIN* (C) and *AhENOD40* (D) transcripts was performed using total RNA samples extracted from control (EV) and *AhERN1*-overexpressing (*35S:AhERN1*) nodules at 28 dpi.

Supplementary Table S1. Primers used in this study.

Primer	Sequence	Application
AhERN1-CDS-F	CACCATGTTCCCGATGGAAATTCATTTCC	For cloning of the *AhERN1* gene full
AhERN1-CDS-R	TTAGCAGAATAAGGAGCACAAAGG	length CDS
AhERN1-Pro-F	CACCGTTCTGAGGACAAAGACATGGCTAC	For cloning of the *AhERN1* gene
AhERN1-Pro-R	AGTATCTGGGGAGTATAGGGTTTAGG	promoter sequence
AhERN1-F1	ATGTTCCCGATGGAAATTCATTTC	For cloning of the AhERN1 protein
AhERN1-R1	CCTCACTCCAACAAATTTGTTTGT	N terminal
AhERN1-F2	AACAAATTTGTTGGAGTGAGGCAG	For cloning of the AhERN1 protein
AhERN1-R2	AGAATCCGTGGAGACATGAGTG	AP2 domain
AhERN1-F3	ACTCATGTCTCCACGGATTCTC	For cloning of the AhERN1 protein
AhERN1-R3	TTAGCAGAATAAGGAGCACAAAGG	C terminal
AhERN1-qRT-F	TTTCGGACCCATCTTCGACC	For RT-qPCR analysis of *AhERN1*
AhERN1-qRT-R	TTGGGCCGCCGGTAATAAG	gene expression
AhNFR5-qRT-F	CTCATTGAGTTGCTGACAGGC	For RT-qPCR analysis of *AhNFR5*
AhNFR5-qRT-R	CCATCTTCTCAGACACTCCTCC	gene expression
AhHK1-qRT-F	GAGGTCTTAAAGCCATTGTGGTTGATGG	For RT-qPCR analysis of *AhCRE1*
AhHK1-qRT-R	CAACTGCTTGATAGAGATTCTCTTC	gene expression
AhNIN-qRT-F	TTTGGGAGCTTGTCGGAGTC	For RT-qPCR analysis of *AhNIN*
AhNIN-qRT-R	CCAGTTGTAGCACCCTCCTG	gene expression
AhENOD16-qRT-F	GGCAAAAATCCATCCATGGTTC	For RT-qPCR analysis of
AhENOD16-qRT-R	GCCTTTTTGTGACTTGCCGG	*AhENOD40* gene expression
AdG6PD-qRT-F	ACCATTCCAGAGGCTTATGAGC	RT-qPCR analysis of internal
AdG6PD-qRT-R	AAGGGAGTGACTTGAACTCTCC	control gene
